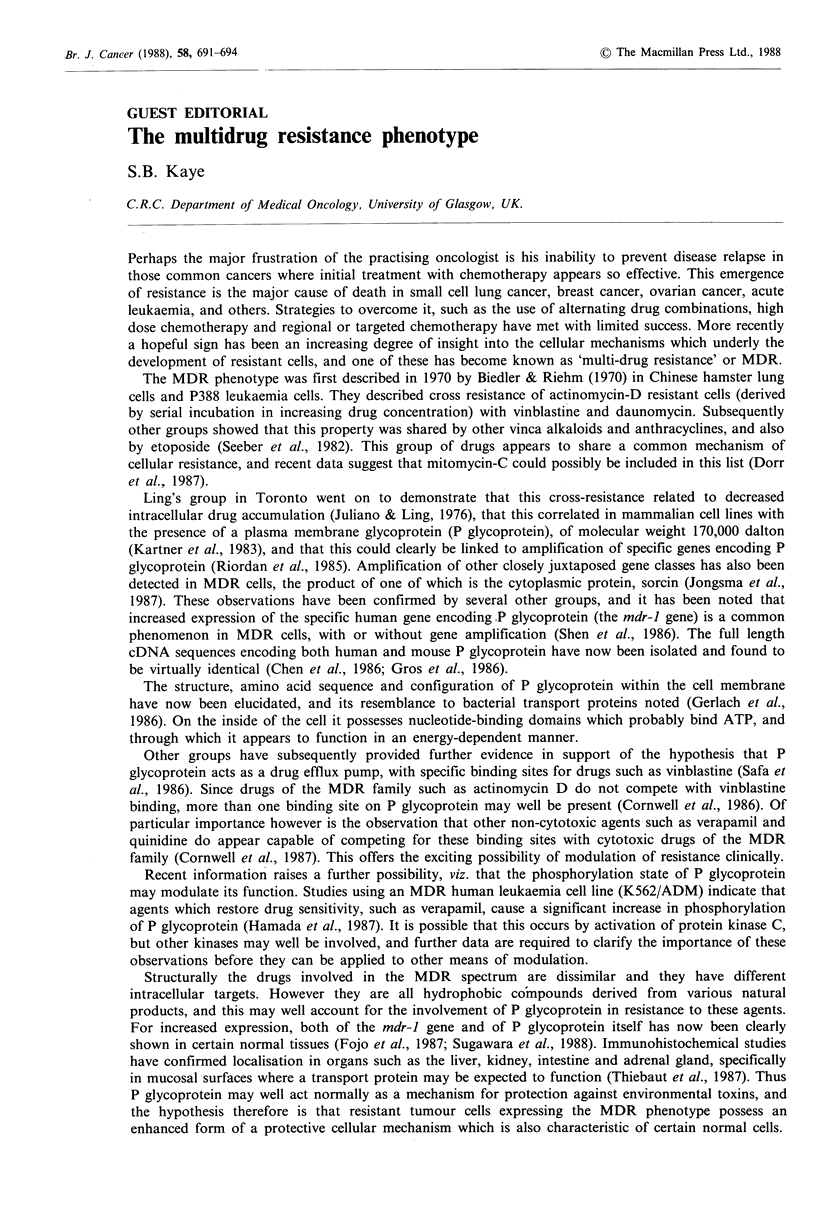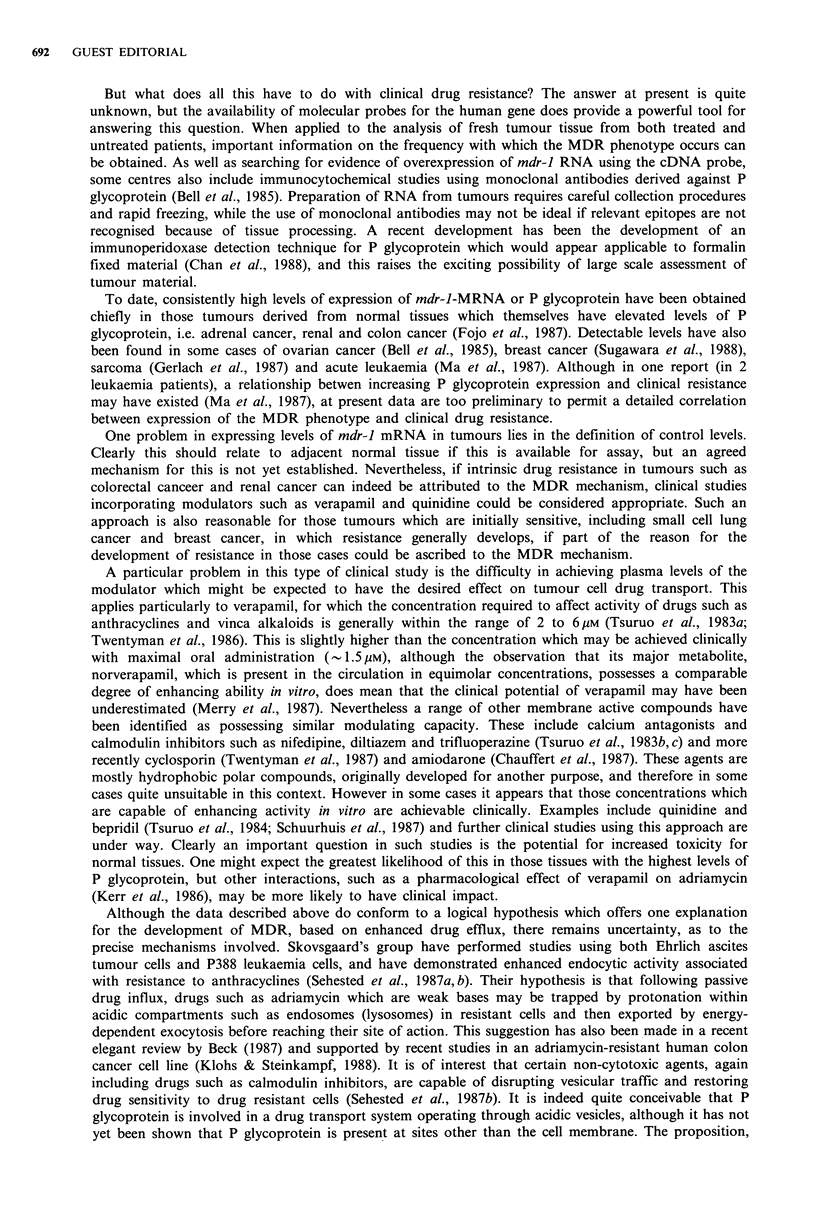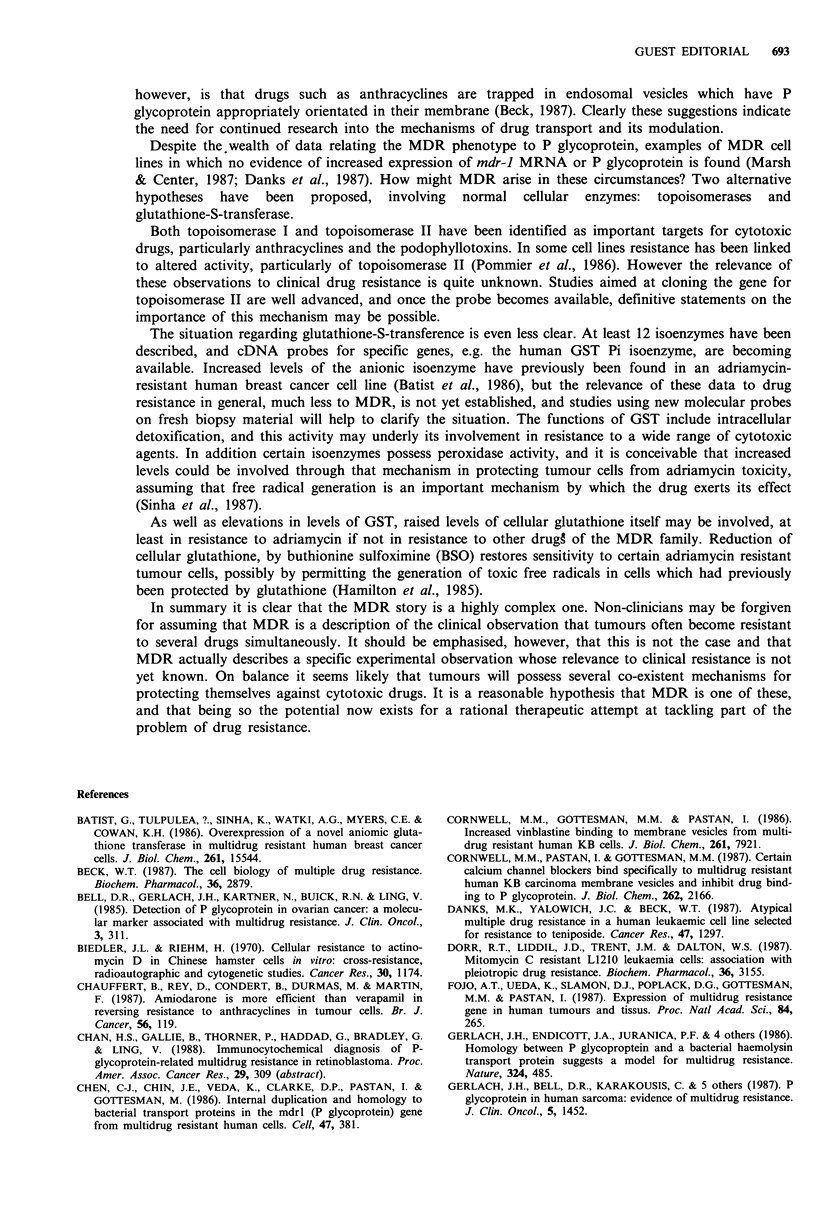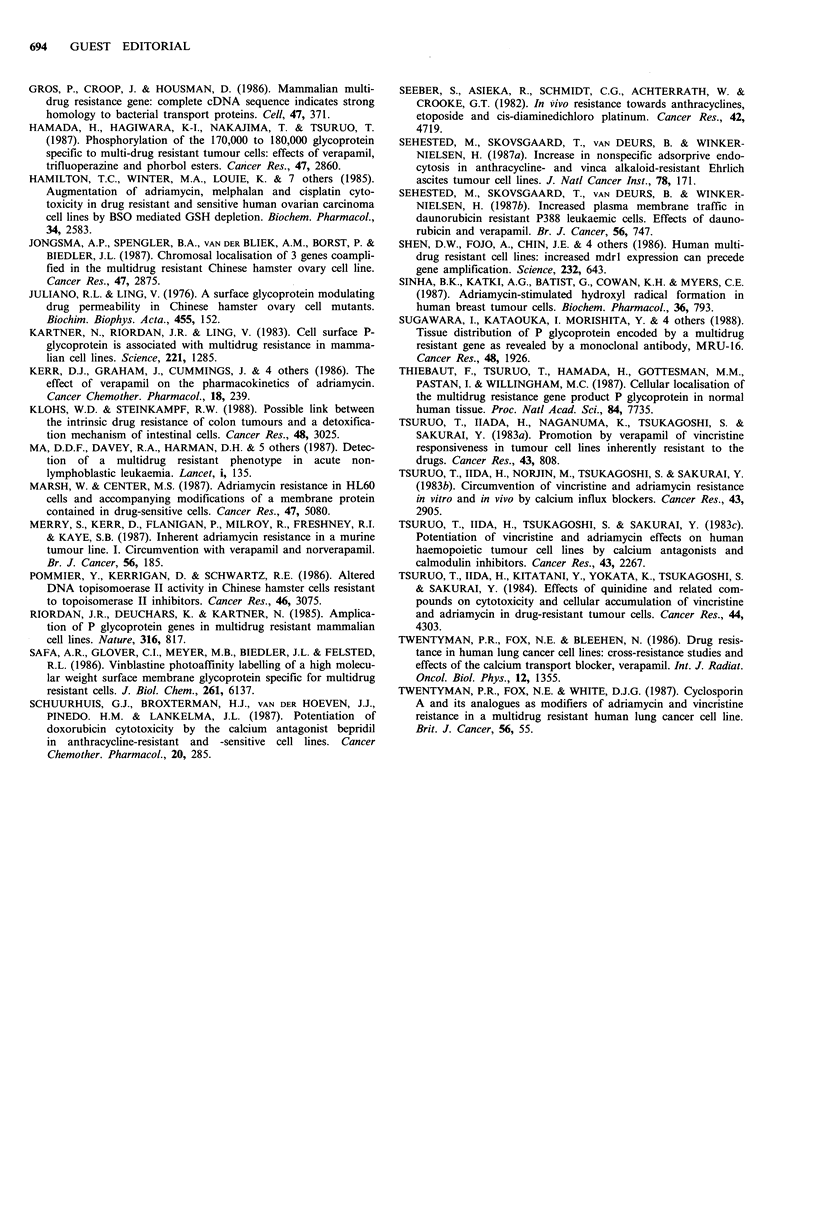# The multidrug resistance phenotype.

**DOI:** 10.1038/bjc.1988.291

**Published:** 1988-12

**Authors:** S. B. Kaye

**Affiliations:** C.R.C. Department of Medical Oncology, University of Glasgow, UK.


Br. J. Cancer (1988), 58, 691–694 © The Macmillan Press Ltd., 1988

GUEST EDITORIAL

### The multidrug resistance phenotype

S.B. Kaye

*C.R.C. Department of Medical Oncology, University of Glasgow, UK.*

Perhaps the major frustration of the practising oncologist is his inability to prevent disease relapse in those common cancers where initial treatment with chemotherapy appears so effective. This emergence of resistance is the major cause of death in small cell lung cancer, breast cancer, ovarian cancer, acute leukaemia, and others. Strategies to overcome it, such as the use of alternating drug combinations, high dose chemotherapy and regional or targeted chemotherapy have met with limited success. More recently a hopeful sign has been an increasing degree of insight into the cellular mechanisms which underly the development of resistant cells, and one of these has become known as 'multi-drug resistance' or MDR.

The MDR phenotype was first described in 1970 by Biedler & Riehm (1970) in Chinese hamster lung cells and P388 leukaemia cells. They described cross resistance of actinomycin-D resistant cells (derived by serial incubation in increasing drug concentration) with vinblastine and daunomycin. Subsequently other groups showed that this property was shared by other vinca alkaloids and anthracyclines, and also by etoposide (Seeber *et al.*, 1982). This group of drugs appears to share a common mechanism of cellular resistance, and recent data suggest that mitomycin-C could possibly be included in this list (Dorr *et al.*, 1987).

Ling's group in Toronto went on to demonstrate that this cross-resistance related to decreased intracellular drug accumulation (Juliano & Ling, 1976), that this correlated in mammalian cell lines with the presence of a plasma membrane glycoprotein (P glycoprotein), of molecular weight 170,000 dalton (Kartner *et al.*, 1983), and that this could clearly be linked to amplification of specific genes encoding P glycoprotein (Riordan *et al.*, 1985). Amplification of other closely juxtaposed gene classes has also been detected in MDR cells, the product of one of which is the cytoplasmic protein, sorcin (Jongsma *et al.*, 1987). These observations have been confirmed by several other groups, and it has been noted that increased expression of the specific human gene encoding P glycoprotein (the *mdr-1* gene) is a common phenomenon in MDR cells, with or without gene amplification (Shen *et al.*, 1986). The full length cDNA sequences encoding both human and mouse P glycoprotein have now been isolated and found to be virtually identical (Chen *et al.*, 1986; Gros *et al.*, 1986).

The structure, amino acid sequence and configuration of P glycoprotein within the cell membrane have now been elucidated, and its resemblance to bacterial transport proteins noted (Gerlach *et al.*, 1986). On the inside of the cell it possesses nucleotide-binding domains which probably bind ATP, and through which it appears to function in an energy-dependent manner.

Other groups have subsequently provided further evidence in support of the hypothesis that P glycoprotein acts as a drug efflux pump, with specific binding sites for drugs such as vinblastine (Safa *et al.*, 1986). Since drugs of the MDR family such as actinomycin D do not compete with vinblastine binding, more than one binding site on P glycoprotein may well be present (Cornwell *et al.*, 1986). Of particular importance however is the observation that other non-cytotoxic agents such as verapamil and quinidine do appear capable of competing for these binding sites with cytotoxic drugs of the MDR family (Cornwell *et al.*, 1987). This offers the exciting possibility of modulation of resistance clinically.

Recent information raises a further possibility, *viz.* that the phosphorylation state of P glycoprotein may modulate its function. Studies using an MDR human leukaemia cell line (K562/ADM) indicate that agents which restore drug sensitivity, such as verapamil, cause a significant increase in phosphorylation of P glycoprotein (Hamada *et al.*, 1987). It is possible that this occurs by activation of protein kinase C, but other kinases may well be involved, and further data are required to clarify the importance of these observations before they can be applied to other means of modulation.

Structurally the drugs involved in the MDR spectrum are dissimilar and they have different intracellular targets. However they are all hydrophobic compounds derived from various natural products, and this may well account for the involvement of P glycoprotein in resistance to these agents. For increased expression, both of the *mdr-1* gene and of P glycoprotein itself has now been clearly shown in certain normal tissues (Fojo *et al.*, 1987; Sugawara *et al.*, 1988). Immunohistochemical studies have confirmed localisation in organs such as the liver, kidney, intestine and adrenal gland, specifically in mucosal surfaces where a transport protein may be expected to function (Thiebaut *et al.*, 1987). Thus P glycoprotein may well act normally as a mechanism for protection against environmental toxins, and the hypothesis therefore is that resistant tumour cells expressing the MDR phenotype possess an enhanced form of a protective cellular mechanism which is also characteristic of certain normal cells.

But what does all this have to do with clinical drug resistance? The answer at present is quite unknown, but the availability of molecular probes for the human gene does provide a powerful tool for answering this question. When applied to the analysis of fresh tumour tissue from both treated and untreated patients, important information on the frequency with which the MDR phenotype occurs can be obtained. As well as searching for evidence of overexpression of *mdr-1* RNA using the cDNA probe, some centres also include immunocytochemical studies using monoclonal antibodies derived against P glycoprotein (Bell *et al.*, 1985). Preparation of RNA from tumours requires careful collection procedures and rapid freezing, while the use of monoclonal antibodies may not be ideal if relevant epitopes are not recognised because of tissue processing. A recent development has been the development of an immunoperidoxase detection technique for P glycoprotein which would appear applicable to formalin fixed material (Chan *et al.*, 1988), and this raises the exciting possibility of large scale assessment of tumour material.

To date, consistently high levels of expression of *mdr-1*-MRNA or P glycoprotein have been obtained chiefly in those tumours derived from normal tissues which themselves have elevated levels of P glycoprotein, i.e. adrenal cancer, renal and colon cancer (Fojo *et al.*, 1987). Detectable levels have also been found in some cases of ovarian cancer (Bell *et al.*, 1985), breast cancer (Sugawara *et al.*, 1988), sarcoma (Gerlach *et al.*, 1987) and acute leukaemia (Ma *et al.*, 1987). Although in one report (in 2 leukaemia patients), a relationship betwen increasing P glycoprotein expression and clinical resistance may have existed (Ma *et al.*, 1987), at present data are too preliminary to permit a detailed correlation between expression of the MDR phenotype and clinical drug resistance.

One problem in expressing levels of *mdr-1* mRNA in tumours lies in the definition of control levels. Clearly this should relate to adjacent normal tissue if this is available for assay, but an agreed mechanism for this is not yet established. Nevertheless, if intrinsic drug resistance in tumours such as colorectal canceer and renal cancer can indeed be attributed to the MDR mechanism, clinical studies incorporating modulators such as verapamil and quinidine could be considered appropriate. Such an approach is also reasonable for those tumours which are initially sensitive, including small cell lung cancer and breast cancer, in which resistance generally develops, if part of the reason for the development of resistance in those cases could be ascribed to the MDR mechanism.

A particular problem in this type of clinical study is the difficulty in achieving plasma levels of the modulator which might be expected to have the desired effect on tumour cell drug transport. This applies particularly to verapamil, for which the concentration required to affect activity of drugs such as anthracyclines and vinca alkaloids is generally within the range of 2 to 6 $\mu$M (Tsuruo *et al.*, 1983*a*; Twentyman *et al.*, 1986). This is slightly higher than the concentration which may be achieved clinically with maximal oral administration ($\sim$1.5 $\mu$M), although the observation that its major metabolite, norverapamil, which is present in the circulation in equimolar concentrations, possesses a comparable degree of enhancing ability *in vitro*, does mean that the clinical potential of verapamil may have been underestimated (Merry *et al.*, 1987). Nevertheless a range of other membrane active compounds have been identified as possessing similar modulating capacity. These include calcium antagonists and calmodulin inhibitors such as nifedipine, diltiazem and trifluoperazine (Tsuruo *et al.*, 1983*b*, *c*) and more recently cyclosporin (Twentyman *et al.*, 1987) and amiodarone (Chauffert *et al.*, 1987). These agents are mostly hydrophobic polar compounds, originally developed for another purpose, and therefore in some cases quite unsuitable in this context. However in some cases it appears that those concentrations which are capable of enhancing activity *in vitro* are achievable clinically. Examples include quinidine and bepridil (Tsuruo *et al.*, 1984; Schuurhuis *et al.*, 1987) and further clinical studies using this approach are under way. Clearly an important question in such studies is the potential for increased toxicity for normal tissues. One might expect the greatest likelihood of this in those tissues with the highest levels of P glycoprotein, but other interactions, such as a pharmacological effect of verapamil on adriamycin (Kerr *et al.*, 1986), may be more likely to have clinical impact.

Although the data described above do conform to a logical hypothesis which offers one explanation for the development of MDR, based on enhanced drug efflux, there remains uncertainty, as to the precise mechanisms involved. Skovsgaard's group have performed studies using both Ehrlich ascites tumour cells and P388 leukaemia cells, and have demonstrated enhanced endocytic activity associated with resistance to anthracyclines (Sehested *et al.*, 1987*a*, *b*). Their hypothesis is that following passive drug influx, drugs such as adriamycin which are weak bases may be trapped by protonation within acidic compartments such as endosomes (lysosomes) in resistant cells and then exported by energy-dependent exocytosis before reaching their site of action. This suggestion has also been made in a recent elegant review by Beck (1987) and supported by recent studies in an adriamycin-resistant human colon cancer cell line (Klohs & Steinkampf, 1988). It is of interest that certain non-cytotoxic agents, again including drugs such as calmodulin inhibitors, are capable of disrupting vesicular traffic and restoring drug sensitivity to drug resistant cells (Sehested *et al.*, 1987*b*). It is indeed quite conceivable that P glycoprotein is involved in a drug transport system operating through acidic vesicles, although it has not yet been shown that P glycoprotein is present at sites other than the cell membrane. The proposition, however, is that drugs such as anthracyclines are trapped in endosomal vesicles which have P glycoprotein appropriately orientated in their membrane (Beck, 1987). Clearly these suggestions indicate the need for continued research into the mechanisms of drug transport and its modulation.

Despite the wealth of data relating the MDR phenotype to P glycoprotein, examples of MDR cell lines in which no evidence of increased expression of *mdr-1* MRNA or P glycoprotein is found (Marsh & Center, 1987; Danks *et al.*, 1987). How might MDR arise in these circumstances? Two alternative hypotheses have been proposed, involving normal cellular enzymes: topoisomerases and glutathione-S-transferase.

Both topoisomerase I and topoisomerase II have been identified as important targets for cytotoxic drugs, particularly anthracyclines and the podophyllotoxins. In some cell lines resistance has been linked to altered activity, particularly of topoisomerase II (Pommier *et al.*, 1986). However the relevance of these observations to clinical drug resistance is quite unknown. Studies aimed at cloning the gene for topoisomerase II are well advanced, and once the probe becomes available, definitive statements on the importance of this mechanism may be possible.

The situation regarding glutathione-S-transference is even less clear. At least 12 isoenzymes have been described, and cDNA probes for specific genes, e.g. the human GST Pi isoenzyme, are becoming available. Increased levels of the anionic isoenzyme have previously been found in an adriamycin-resistant human breast cancer cell line (Batist *et al.*, 1986), but the relevance of these data to drug resistance in general, much less to MDR, is not yet established, and studies using new molecular probes on fresh biopsy material will help to clarify the situation. The functions of GST include intracellular detoxification, and this activity may underly its involvement in resistance to a wide range of cytotoxic agents. In addition certain isoenzymes possess peroxidase activity, and it is conceivable that increased levels could be involved through that mechanism in protecting tumour cells from adriamycin toxicity, assuming that free radical generation is an important mechanism by which the drug exerts its effect (Sinha *et al.*, 1987).

As well as elevations in levels of GST, raised levels of cellular glutathione itself may be involved, at least in resistance to adriamycin if not in resistance to other drugs of the MDR family. Reduction of cellular glutathione, by buthionine sulfoximine (BSO) restores sensitivity to certain adriamycin resistant tumour cells, possibly by permitting the generation of toxic free radicals in cells which had previously been protected by glutathione (Hamilton *et al.*, 1985).

In summary it is clear that the MDR story is a highly complex one. Non-clinicians may be forgiven for assuming that MDR is a description of the clinical observation that tumours often become resistant to several drugs simultaneously. It should be emphasised, however, that this is not the case and that MDR actually describes a specific experimental observation whose relevance to clinical resistance is not yet known. On balance it seems likely that tumours will possess several co-existent mechanisms for protecting themselves against cytotoxic drugs. It is a reasonable hypothesis that MDR is one of these, and that being so the potential now exists for a rational therapeutic attempt at tackling part of the problem of drug resistance.

### References

BATIST, G., TULPULEA, ?., SINHA, K., WATKI, A.G., MYERS, C.E. & COWAN, K.H. (1986). Overexpression of a novel aniomic glutathione transferase in multidrug resistant human breast cancer cells. *J. Biol. Chem.*, **261**, 15544.

BECK, W.T. (1987). The cell biology of multiple drug resistance. *Biochem. Pharmacol.*, **36**, 2879.

BELL, D.R., GERLACH, J.H., KARTNER, N., BUICK, R.N. & LING, V. (1985). Detection of P glycoprotein in ovarian cancer: a molecular marker associated with multidrug resistance. *J. Clin. Oncol.*, **3**, 311.

BIEDLER, J.L. & RIEHM, H. (1970). Cellular resistance to actinomycin D in Chinese hamster cells *in vitro*: cross-resistance, radioautographic and cytogenetic studies. *Cancer Res.*, **30**, 1174.

CHAUFFERT, B., REY, D., CONDERT, B., DURMAS, M. & MARTIN, F. (1987). Amiodarone is more efficient than verapamil in reversing resistance to anthracyclines in tumour cells. *Br. J. Cancer*, **56**, 119.

CHAN, H.S., GALLIE, B., THORNER, P., HADDAD, G., BRADLEY, G. & LING, V. (1988). Immunocytochemical diagnosis of P-glycoprotein-related multidrug resistance in retinoblastoma. *Proc. Amer. Assoc. Cancer Res.*, **29**, 309 (*abstract*).

CHEN, C-J., CHIN, J.E., VEDA, K., CLARKE, D.P., PASTAN, I. & GOTTESMAN, M. (1986). Internal duplication and homology to bacterial transport proteins in the mdrl (P glycoprotein) gene from multidrug resistant human cells. *Cell*, **47**, 381.

CORNWELL, M.M., GOTTESMAN, M.M. & PASTAN, I. (1986). Increased vinblastine binding to membrane vesicles from multidrug resistant human KB cells. *J. Biol. Chem.*, **261**, 7921.

CORNWELL, M.M., PASTAN, I. & GOTTESMAN, M.M. (1987). Certain calcium channel blockers bind specifically to multidrug resistant human KB carcinoma membrane vesicles and inhibit drug binding to P glycoprotein. *J. Biol. Chem.*, **262**, 2166.

DANKS, M.K., YALOWICH, J.C. & BECK, W.T. (1987). Atypical multiple drug resistance in a human leukaemic cell line selected for resistance to teniposide. *Cancer Res.*, **47**, 1297.

DORR, R.T., LIDDIL, J.D., TRENT, J.M. & DALTON, W.S. (1987). Mitomycin C resistant L1210 leukaemia cells: association with pleiotropic drug resistance. *Biochem. Pharmacol.*, **36**, 3155.

FOJO, A.T., UEDA, K., SLAMON, D.J., POPLACK, D.G., GOTTESMAN, M.M. & PASTAN, I. (1987). Expression of multidrug resistance gene in human tumours and tissus. *Proc. Natl Acad. Sci.*, **84**, 265.

GERLACH, J.H., ENDICOTT, J.A., JURANICA, P.F. & 4 others (1986). Homology between P glycoproptein and a bacterial haemolysin transport protein suggests a model for multidrug resistance. *Nature*, **324**, 485.

GERLACH, J.H., BELL, D.R., KARAKOUSIS, C. & 5 others (1987). P glycoprotein in human sarcoma: evidence of multidrug resistance. *J. Clin. Oncol.*, **5**, 1452.

GROS, P., CROOP, J. & HOUSMAN, D. (1986). Mammalian multidrug resistance gene: complete cDNA sequence indicates strong homology to bacterial transport proteins. *Cell*, **47**, 371.

HAMADA, H., HAGIWARA, K-I., NAKAJIMA, T. & TSURUO, T. (1987). Phosphorylation of the 170,000 to 180,000 glycoprotein specific to multi-drug resistant tumour cells: effects of verapamil, trifluoperazine and phorbol esters. *Cancer Res.*, **47**, 2860.

HAMILTON, T.C., WINTER, M.A., LOUIE, K. & 7 others (1985). Augmentation of adriamycin, melphalan and cisplatin cytotoxicity in drug resistant and sensitive human ovarian carcinoma cell lines by BSO mediated GSH depletion. *Biochem. Pharmacol.*, **34**, 2583.

JONGSMA, A.P., SPENGLER, B.A., VAN DER BLIEK, A.M., BORST, P. & BIEDLER, J.L. (1987). Chromosal localisation of 3 genes coamplified in the multidrug resistant Chinese hamster ovary cell line. *Cancer Res.*, **47**, 2875.

JULIANO, R.L. & LING, V. (1976). A surface glycoprotein modulating drug permeability in Chinese hamster ovary cell mutants. *Biochim. Biophys. Acta.*, **455**, 152.

KARTNER, N., RIORDAN, J.R. & LING, V. (1983). Cell surface P-glycoprotein is associated with multidrug resistance in mammalian cell lines. *Science*, **221**, 1285.

KERR, D.J., GRAHAM, J., CUMMINGS, J. & 4 others (1986). The effect of verapamil on the pharmacokinetics of adriamycin. *Cancer Chemother. Pharmacol.*, **18**, 239.

KLOHS, W.D. & STEINKAMPF, R.W. (1988). Possible link between the intrinsic drug resistance of colon tumours and a detoxification mechanism of intestinal cells. *Cancer Res.*, **48**, 3025.

MA, D.D.F., DAVEY, R.A., HARMAN, D.H. & 5 others (1987). Detection of a multidrug resistant phenotype in acute non-lymphoblastic leukaemia. *Lancet*, **i**, 135.

MARSH, W. & CENTER, M.S. (1987). Adriamycin resistance in HL60 cells and accompanying modifications of a membrane protein contained in drug-sensitive cells. *Cancer Res.*, **47**, 5080.

MERRY, S., KERR, D., FLANIGAN, P., MILROY, R., FRESHNEY, R.I. & KAYE, S.B. (1987). Inherent adriamycin resistance in a murine tumour line. I. Circumvention with verapamil and norverapamil. *Br. J. Cancer*, **56**, 185.

POMMIER, Y., KERRIGAN, D. & SCHWARTZ, R.E. (1986). Altered DNA topisomoerase II activity in Chinese hamster cells resistant to topoisomerase II inhibitors. *Cancer Res.*, **46**, 3075.

RIORDAN, J.R., DEUCHARS, K. & KARTNER, N. (1985). Amplication of P glycoprotein genes in multidrug resistant mammalian cell lines. *Nature*, **316**, 817.

SAFA, A.R., GLOVER, C.I., MEYER, M.B., BIEDLER, J.L. & FELSTED, R.L. (1986). Vinblastine photoaffinity labelling of a high molecular weight surface membrane glycoprotein specific for multidrug resistant cells. *J. Biol. Chem.*, **261**, 6137.

SCHUURHUIS, G.J., BROXTERMAN, H.J., VAN DER HOEVEN, J.J., PINEDO. H.M. & LANKELMA, J.L. (1987). Potentiation of doxorubicin cytotoxicity by the calcium antagonist bepridil in anthracycline-resistant and -sensitive cell lines. *Cancer Chemother. Pharmacol.*, **20**, 285.

SEEBER, S., ASIEKA, R., SCHMIDT, C.G., ACHTERRATH, W. & CROOKE, G.T. (1982). *In vivo* resistance towards anthracyclines, etoposide and cis-diaminedichloro platinum. *Cancer Res.*, **42**, 4719.

SEHESTED, M., SKOVSGAARD, T., VAN DEURS, B. & WINKER-NIELSEN, H. (1987*a*). Increase in nonspecific adsorprive endocytosis in anthracycline- and vinca alkaloid-resistant Ehrlich ascites tumour cell lines. *J. Natl Cancer Inst.*, **78**, 171.

SEHESTED, M., SKOVSGAARD, T., VAN DEURS, B. & WINKER-NIELSEN, H. (1987*b*). Increased plasma membrane traffic in daunorubicin resistant P388 leukaemic cells. Effects of daunorubicin and verapamil. *Br. J. Cancer*, **56**, 747.

SHEN, D.W., FOJO, A., CHIN, J.E. & 4 others (1986). Human multidrug resistant cell lines: increased mdr1 expression can precede gene amplification. *Science*, **232**, 643.

SINHA, B.K., KATKI, A.G., BATIST, G., COWAN, K.H. & MYERS, C.E. (1987). Adriamycin-stimulated hydroxyl radical formation in human breast tumour cells. *Biochem. Pharmacol.*, **36**, 793.

SUGAWARA, I., KATAOUKA, I. MORISHITA, Y. & 4 others (1988). Tissue distribution of P glycoprotein encoded by a multidrug resistant gene as revealed by a monoclonal antibody, MRU-16. *Cancer Res.*, **48**, 1926.

THIEBAUT, F., TSURUO, T., HAMADA, H., GOTTESMAN, M.M., PASTAN, I. & WILLINGHAM, M.C. (1987). Cellular localisation of the multidrug resistance gene product P glycoprotein in normal human tissue. *Proc. Natl Acad. Sci.*, **84**, 7735.

TSURUO, T., IIADA, H., NAGANUMA, K., TSUKAGOSHI, S. & SAKURAI, Y. (1983*a*). Promotion by verapamil of vincristine responsiveness in tumour cell lines inherently resistant to the drugs. *Cancer Res.*, **43**, 808.

TSURUO, T., IIDA, H., NORJIN, M., TSUKAGOSHI, S. & SAKURAI, Y. (1983*b*). Circumvention of vincristine and adriamycin resistance *in vitro* and *in vivo* by calcium influx blockers. *Cancer Res.*, **43**, 2905.

TSURUO, T., IIDA, H., TSUKAGOSHI, S. & SAKURAI, Y. (1983*c*). Potentiation of vincristine and adriamycin effects on human haemopoietic tumour cell lines by calcium antagonists and calmodulin inhibitors. *Cancer Res.*, **43**, 2267.

TSURUO, T., IIDA, H., KITATANI, Y., YOKATA, K., TSUKAGOSHI, S. & SAKURAI, Y. (1984). Effects of quinidine and related compounds on cytotoxicity and cellular accumulation of vincristine and adriamycin in drug-resistant tumour cells. *Cancer Res.*, **44**, 4303.

TWENTYMAN, P.R., FOX, N.E. & BLEEHEN, N. (1986). Drug resistance in human lung cancer cell lines: cross-resistance studies and effects of the calcium transport blocker, verapamil. *Int. J. Radiat. Oncol. Biol. Phys.*, **12**, 1355.

TWENTYMAN, P.R., FOX, N.E. & WHITE, D.J.G. (1987). Cyclosporin A and its analogues as modifiers of adriamycin and vincristine resistance in a multidrug resistant human lung cancer cell line. *Brit. J. Cancer*, **56**, 55.